# (*S*,*Z*)-1-Chloro-3-[(3,4,5-trimeth­oxy­benzyl­idene)amino]­propan-2-ol

**DOI:** 10.1107/S1600536810053420

**Published:** 2011-01-08

**Authors:** Yun Ren, Shan Qian, Li Hai, Wei Fan, Yong Wu

**Affiliations:** a Key Laboratory of Drug Targeting of the Education Ministry, West China School of Pharmacy, Sichuan University, Chengdu 610041, People’s Republic of China; b Bioengineering College, Xihua University, Chengdu 610039, People’s Republic of China

## Abstract

In the title compound, C_13_H_18_ClNO_4_, the two meth­oxy groups at the *meta* positions of the attached benzene ring are close to being coplanar with the ring [the meth­oxy C atoms deviate by 0.267 (7) and 0.059 (7) Å], whereas the third meth­oxy group at the *para* position is not coplanar with the benzene ring [methoxy C atom deviates by 1.100 (6) Å]. In the crystal, mol­ecules are linked into a chain along the *a* axis by O—H⋯N hydrogen bonds.

## Related literature

The title compound is an inter­mediate for the synthesis of linezolid [systematic name (*S*)-*N*{3-[3-fluoro-4-(morpholin-4-yl)phen­yl]-2-oxo-1,3-oxazolidin-5-yl}meth­yl)acetamide], which is currently used in the treatment of serious multi-drug resistant Gram-positive bacterial infections caused by strains of staphylococci, streptococci and enterococci, see: Brickner *et al.* (1996 ▶); Perrault *et al.* (2002 ▶). For synthetic procedures, see: Imbordino *et al.* (2007 ▶); Zhao *et al.* (2006 ▶).
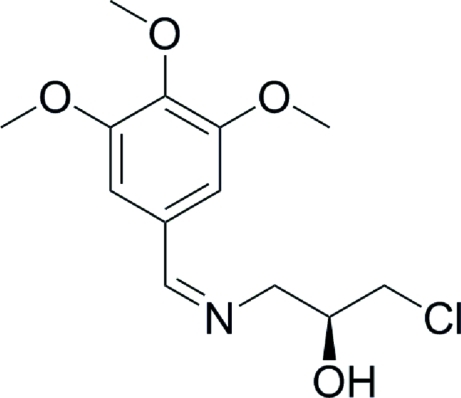


## Experimental

### 

#### Crystal data

C_13_H_18_ClNO_4_
*M* *_r_* = 287.73
Orthorhombic, 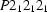

*a* = 6.5332 (12) Å
*b* = 8.888 (2) Å
*c* = 25.29 (3) Å
*V* = 1468.7 (16) Å^3^
*Z* = 4
Mo *K*α radiation
μ = 0.27 mm^−1^
*T* = 296 K
0.32 × 0.28 × 0.20 mm

#### Data collection

Xcalibur, Eos diffractometer
Absorption correction: multi-scan (*CrysAlis PRO*; Oxford Diffraction 2009 ▶) *T*_min_ = 0.760, *T*_max_ = 1.0
3967 measured reflections
2695 independent reflections
1599 reflections with *I* > 2σ(*I*)
*R*_int_ = 0.045

#### Refinement

*R*[*F* ^2^ > 2σ(*F* ^2^)] = 0.051
*wR*(*F* ^2^) = 0.126
*S* = 1.06
2695 reflections
176 parameters
H-atom parameters constrained
Δρ_max_ = 0.32 e Å^−3^
Δρ_min_ = −0.21 e Å^−3^
Absolute structure: Flack (1983 ▶), 931 Friedel pairs
Flack parameter: 0.18 (13)

### 

Data collection: *CrysAlis PRO* (Oxford Diffraction, 2009 ▶); cell refinement: *CrysAlis PRO*; data reduction: *CrysAlis PRO*; program(s) used to solve structure: *SHELXS97* (Sheldrick, 2008 ▶); program(s) used to refine structure: *SHELXL97* (Sheldrick, 2008 ▶); molecular graphics: *OLEX2* (Dolomanov *et al.*, 2009 ▶); software used to prepare material for publication: *OLEX2*.

## Supplementary Material

Crystal structure: contains datablocks global, I. DOI: 10.1107/S1600536810053420/rn2076sup1.cif

Structure factors: contains datablocks I. DOI: 10.1107/S1600536810053420/rn2076Isup2.hkl

Additional supplementary materials:  crystallographic information; 3D view; checkCIF report

## Figures and Tables

**Table 1:** Hydrogen-bond geometry (Å, °)

*D*—H⋯*A*	*D*—H	H⋯*A*	*D*⋯*A*	*D*—H⋯*A*
O1—H1⋯N1^i^	0.82	2.08	2.870 (4)	162

Symmetry code: (i) 
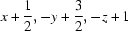
.

**Fig. 1.**
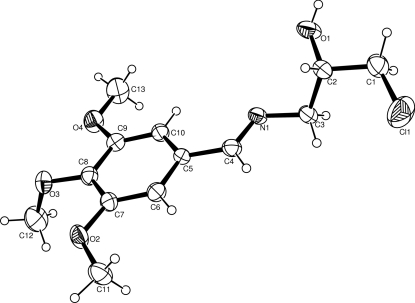
The molecular structure of the title compound, with displacement ellipsoids drawn at the 50% probability level.

**Fig. 2.**
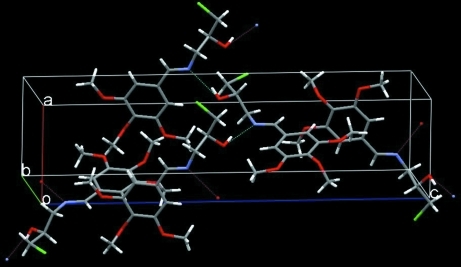
A packing diagram for the title compound.

## supplementary crystallographic information

### Comment

The optically active *S,Z*-1-chloro-3-(3,4,5-trimethoxybenzylideneamino) propan-2-ol is a key intermediate for synthesizing Linezolid. Linezolid is a potent, synthetic oxazolidinone, which is currently used in the treatment of serious multi-drug resistant Gram-positive bacterial infections caused by strains of staphylococci, streptococci, and enterococci (Brickner *et al.*, 1996; Perrault *et al.*, 2002). Our interests in synthesizing Linezolid prompted us to develop an efficient methodology for synthesizing *S,Z*-1-chloro-3-(3,4,5-trimethoxybenzylideneamino)propan-2-ol. In our synthetic work, we obtained the title compound, whose spectral data corresponds with that reported in the literature (Imbordino *et al.*, 2007; Zhao *et al.*, 2006). Its crystal structure is reported here. The two methoxy groups at the *meta* positions are approximately coplanar with the attached benzene ring, and the *C*(methoxy) atoms, C11 and C13, are -0.2672 (65) and -0.0588 (73) Å from the plane of benzene ring. Whereas the third methoxy group at the *para* position is not coplanar with the ring, and the distance of the *C*(methoxy) atom, C12, is -1.1003 (64) Å. An intermolecular O—H···N hydrogen bond is observed. The molecules are linked into a chain along the *a* axis by O—H···N hydrogen bonds.

### Experimental

To a stirred solution of 3,4,5-trimethoxybenzaldehyde (20.0 g,102 mmol) in 200 ml of methyl *tert*-butyl ether at room temperature was added concentrated ammonia water (12 ml,161 mmol). After 1 h, to this stirred solution at room temperature was added, dropwise over 20 min, the solution of *S*-2-(chloromethyl)oxirane (8 ml, 102 mmol) in 200 ml of methyl *tert*-butyl ether. After 24 h, the organic layer was separated and dried (MgSO_4_) and then concentrated under reduced pressure. The residue is dispersed in methyl *tert*-butyl ether, and left to crystallize 17.3 g (yield 58.8%) of *S,Z*-1-chloro-3-(3,4,5-trimethoxybenzylideneamino)propan-2-ol. Colourless crystals suitable for X-ray analysis were obtained by slow evaporation in methanol at room temperature.

### Refinement

All H atoms were positioned geometrically (C—H = 0.93–0.96 Å) and refined using a riding model, with *U*_iso_(H) = 1.2–1.5*U*_eq_(C).

### Crystal data

**Table:**

C_13_H_18_ClNO_4_	*F*(000) = 608
*M**_r_* = 287.73	*D*_x_ = 1.301 Mg m^−^^3^
Orthorhombic, *P*2_1_2_1_2_1_	Mo *K*α radiation, λ = 0.7107 Å
Hall symbol: P 2ac 2ab	Cell parameters from 699 reflections
*a* = 6.5332 (12) Å	θ = 3.1–29.2°
*b* = 8.888 (2) Å	µ = 0.27 mm^−^^1^
*c* = 25.29 (3) Å	*T* = 296 K
*V* = 1468.7 (16) Å^3^	Block, colourless
*Z* = 4	0.32 × 0.28 × 0.20 mm

### Data collection

**Table:**

Xcalibur, Eos diffractometer	2695 independent reflections
Radiation source: Enhance (Mo) X-ray Source	1599 reflections with *I* > 2σ(*I*)
graphite	*R*_int_ = 0.045
Detector resolution: 16.0874 pixels mm^-1^	θ_max_ = 26.4°, θ_min_ = 3.2°
ω scans	*h* = −8→8
Absorption correction: multi-scan (*CrysAlis PRO*; Oxford Diffraction 2009)	*k* = −11→6
*T*_min_ = 0.760, *T*_max_ = 1.0	*l* = −20→31
3967 measured reflections	

### Refinement

**Table:**

Refinement on *F*^2^	Secondary atom site location: difference Fourier map
Least-squares matrix: full	Hydrogen site location: inferred from neighbouring sites
*R*[*F*^2^ > 2σ(*F*^2^)] = 0.051	H-atom parameters constrained
*wR*(*F*^2^) = 0.126	*w* = 1/[σ^2^(*F*_o_^2^) + (0.0534*P*)^2^] where *P* = (*F*_o_^2^ + 2*F*_c_^2^)/3
*S* = 1.06	(Δ/σ)_max_ < 0.001
2695 reflections	Δρ_max_ = 0.32 e Å^−^^3^
176 parameters	Δρ_min_ = −0.21 e Å^−^^3^
0 restraints	Absolute structure: Flack (1983), 931 Friedel pairs
Primary atom site location: structure-invariant direct methods	Flack parameter: 0.18 (13)

### Special details

**Table:**

Geometry. All e.s.d.'s (except the e.s.d. in the dihedral angle between two l.s. planes) are estimated using the full covariance matrix. The cell e.s.d.'s are taken into account individually in the estimation of e.s.d.'s in distances, angles and torsion angles; correlations between e.s.d.'s in cell parameters are only used when they are defined by crystal symmetry. An approximate (isotropic) treatment of cell e.s.d.'s is used for estimating e.s.d.'s involving l.s. planes.
Refinement. Refinement of *F*^2^ against ALL reflections. The weighted *R*-factor *wR* and goodness of fit *S* are based on *F*^2^, conventional *R*-factors *R* are based on *F*, with *F* set to zero for negative *F*^2^. The threshold expression of *F*^2^ > σ(*F*^2^) is used only for calculating *R*-factors(gt) *etc*. and is not relevant to the choice of reflections for refinement. *R*-factors based on *F*^2^ are statistically about twice as large as those based on *F*, and *R*- factors based on ALL data will be even larger.

### Fractional atomic coordinates and isotropic or equivalent isotropic displacement parameters (Å^2^)

**Table:**

	*x*	*y*	*z*	*U*_iso_*/*U*_eq_	
Cl1	1.17509 (19)	0.37018 (14)	0.54369 (7)	0.1140 (6)	
O1	0.8309 (4)	0.7058 (3)	0.49115 (10)	0.0664 (7)	
H1	0.9264	0.7436	0.4749	0.100*	
O4	0.0299 (4)	0.9974 (3)	0.65900 (10)	0.0669 (8)	
O2	0.3609 (4)	0.7987 (3)	0.80796 (10)	0.0644 (7)	
O3	0.0912 (4)	0.9855 (3)	0.76403 (10)	0.0617 (7)	
N1	0.5915 (4)	0.6334 (3)	0.58064 (11)	0.0465 (7)	
C1	1.0430 (6)	0.4919 (4)	0.49923 (16)	0.0734 (12)	
H1B	1.1417	0.5473	0.4782	0.088*	
H1A	0.9599	0.4322	0.4754	0.088*	
C2	0.9080 (5)	0.6012 (4)	0.52862 (13)	0.0488 (8)	
H2	0.9893	0.6546	0.5553	0.059*	
C3	0.7283 (5)	0.5258 (4)	0.55492 (15)	0.0522 (9)	
H3A	0.6519	0.4696	0.5286	0.063*	
H3B	0.7783	0.4549	0.5811	0.063*	
C4	0.5835 (5)	0.6308 (4)	0.63124 (14)	0.0488 (9)	
H4	0.6702	0.5640	0.6485	0.059*	
C5	0.4496 (5)	0.7237 (3)	0.66425 (13)	0.0395 (8)	
C6	0.4721 (5)	0.7140 (4)	0.71966 (13)	0.0484 (9)	
H6	0.5707	0.6503	0.7339	0.058*	
C7	0.3478 (5)	0.7990 (4)	0.75334 (14)	0.0472 (9)	
C8	0.2026 (5)	0.8932 (3)	0.73163 (14)	0.0465 (8)	
C9	0.1786 (5)	0.9004 (4)	0.67608 (14)	0.0466 (8)	
C10	0.3006 (5)	0.8172 (4)	0.64293 (14)	0.0467 (9)	
H10	0.2835	0.8234	0.6065	0.056*	
C11	0.4771 (6)	0.6841 (5)	0.83311 (14)	0.0685 (11)	
H11C	0.4668	0.6952	0.8708	0.103*	
H11B	0.6179	0.6924	0.8226	0.103*	
H11A	0.4251	0.5874	0.8229	0.103*	
C12	−0.0824 (6)	0.9189 (5)	0.78748 (19)	0.0875 (15)	
H12C	−0.1499	0.9913	0.8096	0.131*	
H12B	−0.0407	0.8342	0.8084	0.131*	
H12A	−0.1748	0.8859	0.7603	0.131*	
C13	−0.0084 (8)	1.0060 (6)	0.60329 (18)	0.0896 (15)	
H13C	−0.1008	1.0875	0.5962	0.134*	
H13A	−0.0682	0.9133	0.5914	0.134*	
H13B	0.1181	1.0230	0.5849	0.134*	

### Atomic displacement parameters (Å^2^)

**Table:**

	*U*^11^	*U*^22^	*U*^33^	*U*^12^	*U*^13^	*U*^23^
Cl1	0.0817 (7)	0.0840 (8)	0.1764 (16)	0.0268 (7)	0.0172 (9)	0.0122 (9)
O1	0.0611 (14)	0.0825 (17)	0.0555 (17)	0.0065 (15)	0.0092 (14)	0.0266 (15)
O4	0.0658 (15)	0.0730 (18)	0.0620 (19)	0.0251 (14)	−0.0078 (15)	0.0007 (15)
O2	0.0800 (18)	0.0735 (16)	0.0397 (16)	0.0194 (15)	−0.0047 (14)	−0.0117 (13)
O3	0.0610 (15)	0.0594 (15)	0.0647 (17)	0.0101 (14)	0.0073 (14)	−0.0150 (13)
N1	0.0467 (14)	0.0528 (16)	0.0401 (18)	0.0021 (15)	0.0038 (14)	0.0074 (14)
C1	0.067 (2)	0.070 (3)	0.083 (3)	−0.005 (2)	0.023 (2)	−0.007 (2)
C2	0.0478 (17)	0.057 (2)	0.042 (2)	−0.0037 (17)	0.0064 (17)	−0.0005 (18)
C3	0.055 (2)	0.055 (2)	0.046 (2)	−0.0042 (18)	0.0125 (18)	0.0002 (18)
C4	0.0481 (18)	0.051 (2)	0.047 (2)	0.0039 (18)	0.0028 (18)	0.0086 (18)
C5	0.0405 (17)	0.0397 (17)	0.038 (2)	−0.0011 (15)	0.0052 (16)	0.0014 (15)
C6	0.0463 (18)	0.0483 (18)	0.051 (2)	0.0055 (17)	−0.0026 (18)	0.0024 (18)
C7	0.054 (2)	0.0466 (18)	0.041 (2)	−0.0003 (18)	−0.0017 (18)	−0.0044 (17)
C8	0.0449 (17)	0.0445 (18)	0.050 (2)	−0.0021 (18)	0.0023 (18)	−0.0055 (18)
C9	0.0443 (17)	0.0461 (19)	0.049 (2)	−0.0012 (19)	−0.0062 (18)	0.0010 (17)
C10	0.0470 (17)	0.0483 (18)	0.045 (2)	0.0022 (17)	0.0018 (17)	0.0001 (17)
C11	0.072 (2)	0.087 (3)	0.047 (2)	0.008 (2)	−0.002 (2)	0.007 (2)
C12	0.060 (2)	0.103 (3)	0.100 (4)	0.011 (2)	0.022 (3)	−0.005 (3)
C13	0.094 (3)	0.104 (4)	0.071 (3)	0.039 (3)	−0.022 (3)	0.002 (3)

### Geometric parameters (Å, °)

**Table:**

Cl1—C1	1.783 (4)	C4—C5	1.464 (5)
O1—H1	0.8200	C5—C6	1.412 (5)
O1—C2	1.420 (4)	C5—C10	1.389 (5)
O4—C9	1.369 (4)	C6—H6	0.9300
O4—C13	1.433 (5)	C6—C7	1.398 (5)
O2—C7	1.384 (4)	C7—C8	1.379 (5)
O2—C11	1.420 (4)	C8—C9	1.415 (5)
O3—C8	1.369 (4)	C9—C10	1.373 (5)
O3—C12	1.410 (4)	C10—H10	0.9300
N1—C3	1.462 (4)	C11—H11C	0.9600
N1—C4	1.281 (4)	C11—H11B	0.9600
C1—H1B	0.9700	C11—H11A	0.9600
C1—H1A	0.9700	C12—H12C	0.9600
C1—C2	1.508 (5)	C12—H12B	0.9600
C2—H2	0.9800	C12—H12A	0.9600
C2—C3	1.507 (4)	C13—H13C	0.9600
C3—H3A	0.9700	C13—H13A	0.9600
C3—H3B	0.9700	C13—H13B	0.9600
C4—H4	0.9300		
			
Cl1—C1—H1B	109.4	C2—C3—H3B	109.1
Cl1—C1—H1A	109.4	C3—C2—C1	112.7 (3)
O1—C2—C1	107.5 (3)	C3—C2—H2	109.5
O1—C2—H2	109.5	H3A—C3—H3B	107.8
O1—C2—C3	108.0 (3)	C4—N1—C3	117.2 (3)
O4—C9—C8	114.9 (3)	C5—C4—H4	117.1
O4—C9—C10	123.9 (3)	C5—C6—H6	119.6
O4—C13—H13C	109.5	C5—C10—H10	120.3
O4—C13—H13A	109.5	C6—C5—C4	118.0 (3)
O4—C13—H13B	109.5	C7—O2—C11	118.8 (3)
O2—C7—C6	124.8 (3)	C7—C6—C5	120.7 (3)
O2—C11—H11C	109.5	C7—C6—H6	119.6
O2—C11—H11B	109.5	C7—C8—C9	119.9 (3)
O2—C11—H11A	109.5	C8—O3—C12	115.3 (3)
O3—C8—C7	119.4 (3)	C8—C7—O2	116.2 (3)
O3—C8—C9	120.6 (3)	C8—C7—C6	119.0 (3)
O3—C12—H12C	109.5	C9—O4—C13	117.9 (3)
O3—C12—H12B	109.5	C9—C10—C5	119.5 (3)
O3—C12—H12A	109.5	C9—C10—H10	120.3
N1—C3—C2	112.5 (3)	C10—C5—C4	122.3 (3)
N1—C3—H3A	109.1	C10—C5—C6	119.7 (3)
N1—C3—H3B	109.1	C10—C9—C8	121.2 (3)
N1—C4—H4	117.1	H11C—C11—H11B	109.5
N1—C4—C5	125.7 (3)	H11C—C11—H11A	109.5
C1—C2—H2	109.5	H11B—C11—H11A	109.5
H1B—C1—H1A	108.0	H12C—C12—H12B	109.5
C2—O1—H1	109.5	H12C—C12—H12A	109.5
C2—C1—Cl1	111.3 (3)	H12B—C12—H12A	109.5
C2—C1—H1B	109.4	H13C—C13—H13A	109.5
C2—C1—H1A	109.4	H13C—C13—H13B	109.5
C2—C3—H3A	109.1	H13A—C13—H13B	109.5
			
Cl1—C1—C2—O1	172.3 (2)	C5—C6—C7—O2	−178.8 (3)
Cl1—C1—C2—C3	−68.8 (4)	C5—C6—C7—C8	−0.2 (5)
O1—C2—C3—N1	−57.9 (4)	C6—C5—C10—C9	0.8 (5)
O4—C9—C10—C5	178.9 (3)	C6—C7—C8—O3	−174.9 (3)
O2—C7—C8—O3	3.9 (4)	C6—C7—C8—C9	1.3 (5)
O2—C7—C8—C9	−180.0 (3)	C7—C8—C9—O4	179.9 (3)
O3—C8—C9—O4	−4.0 (4)	C7—C8—C9—C10	−1.4 (5)
O3—C8—C9—C10	174.8 (3)	C8—C9—C10—C5	0.3 (5)
N1—C4—C5—C6	−175.3 (3)	C10—C5—C6—C7	−0.9 (5)
N1—C4—C5—C10	6.1 (5)	C11—O2—C7—C6	−13.6 (5)
C1—C2—C3—N1	−176.5 (3)	C11—O2—C7—C8	167.7 (3)
C3—N1—C4—C5	−176.9 (3)	C12—O3—C8—C7	−84.2 (4)
C4—N1—C3—C2	−111.9 (3)	C12—O3—C8—C9	99.6 (4)
C4—C5—C6—C7	−179.5 (3)	C13—O4—C9—C8	−177.6 (4)
C4—C5—C10—C9	179.4 (3)	C13—O4—C9—C10	3.7 (5)

### Hydrogen-bond geometry (Å, °)

**Table:**

*D*—H···*A*	*D*—H	H···*A*	*D*···*A*	*D*—H···*A*
O1—H1···N1^i^	0.82	2.08	2.870 (4)	162

Symmetry codes: (i) *x*+1/2, −*y*+3/2, −*z*+1.
